# The Health Tourists’ Satisfaction Level of Services Provided: A Cross-Sectional Study in Iran

**DOI:** 10.5539/gjhs.v8n9p294

**Published:** 2015-01-31

**Authors:** Ali Mohammad Varzi, Koroush Saki, Khalil Momeni, Ghasem Rajabi Vasokolaei, Zahra Khodakaramifard, Morteza Arab Zouzani, Habib Jalilian

**Affiliations:** 1 Department of Immunology, Faculty of Medicine, Lorestan University of Medical Sciences, Khorramabad, Iran; 2 Department of Physchology, Faculty of Medicine, Lorestan University of Medical Sciences, Khorramabad, Iran; 3 Department of Physchology, Faculty of Medicine, Shahid Beheshti University of Medical Sciences, Tehran, Iran; 4 Iranian Center of Excellence in Health Management, Tabriz University of Medical Sciences, Tabriz, Iran; 5 Research Center for Modeling in Health, Institute for Futures Studies in Health, Kerman University of Medical, Sciences, Kerman, Iran; 6 Department of Health Management and Economics, School of Public Health, Tehran University of Medical Sciences, Tehran, Iran; 7 Research Evaluation Superintendent, Lorestan University of Medical Sciences, Khorramabad, Iran

**Keywords:** satisfaction, health tourist, hospital

## Abstract

**Introduction:**

Patient satisfaction with provided services is used as an indicator of health care quality. Patient satisfaction is defined as patient perception of provided care compared to expected care. This study was administered to evaluate the health tourists’ satisfaction of provided services in Lorestan University of Medical Sciences affiliated hospitals in 2015.

**Method:**

In this descriptive case study, 1800 (696 (54.4%) men and 812 (45.6%) women, 74.5 province native) patients were selected by random sampling from among the patients of Lorestan University of Medical Sciences affiliated hospitals in 2015 spring. The data collection instrument is a semi-structured questionnaire in this study. The questionnaire has 62 general and specific items. Each of the specific items is scaled on four points; satisfied, fairly satisfied, dissatisfied and O.K. In order to analyze the data both descriptive and inferential statistics were used.

**Results:**

Poldokhtar Imam Khomeini Hospital had the highest Level of satisfaction of 68 percent in all aspects (hoteling, discharge, paramedical, nurses, medical and admission) among the studied hospitals. Kuhdasht Imam Khomeini hospital had the lowest level of satisfaction of 53 percent. The overall satisfaction level in all hospitals was 61%.

**Discussion and Conclusion:**

Despite the shortcomings observed in different areas, the results of the present study are in an intermediate status compared to other studies. While treating patients, patient-centered issue and patients ‘need and preferences should be focused on to enhance health care quality. Considering Patients preferences not only are morally good but also lead to improved care and access to sustainable care practices. Therefore it is needed to drive organizational management approach toward the customer preferences management and needs.

## 1. Introduction

Tourism can be considered as an important factor in each country’s economy dynamism and sustainable development. Tourism is one of the largest and most diverse industries in the world. This dynamic industry is known as the main source of income, employment, private sector growth and infrastructure sector development in many countries (Mustafa, 2010). Today, tourism is one of the few service sectors which has provided business opportunities for all nations regardless of their levels of development. This delicate point has resulted in the growth of tourism so that tourism has been interpreted as an “industry” (Hämäläinen et al., 2006). Health tourism means the individual’s structured and systematic trip from one environment to another in order to recover and regain physical and mental health. In recent decades this branch of tourism has developed significantly. One of the main objectives of medical tourism is traveling for treatment and physical and psychological recovery. Often leisure activities are also added to the ill health package in addition to health care services, (Smith & Puczkó, 2008). Patients are forced to receive services in other places due to the differences in the level of cities and countries on one hand, and the health services prices and the provided facilities level on the other hand. In fact, different aspects affect individuals’ demand for a medical trip including health, consumers values change, services price, quality, availability, timeliness, new attitudes toward mental and spiritual activities, treatment without being insured (Kazemi, 2007; Voss et al., 2007). Patient satisfaction as a key factor of health services quality, an important criteria for performance-based payment, and a health policy is emerging worldwide (Leino-Kilpi & Vuorenheimo, 1992; Smith & Englelbrecht, 2001). During the past three decades, paying attention to patient satisfaction with provided services in health centers has become significantly important because of doctors roles change, care change toward patient-centered care and customers attitude change toward the provision of medical and sanitary services (Hawthorne et al., 2014). There are some contradictions in the principles and fundamentals of patient satisfaction despite the increased popularity of its value. The major literature of patient satisfaction with health care services is formed in the 1980s. Patient satisfaction is a challenging and multi-faceted concept (Ware et al., 1983). Marketing theorists have proposed different definitions for satisfaction concept. Cutler defines customer satisfaction as the degree to which actual performance of a company meets customer expectations. In other terms customer satisfaction is a customer’s emotion or attitude toward a product or service (Kotler et al., 1999). Satisfaction is not a predetermined concept but simply and practically it is satisfaction with a level of achieved objectives (Doherty, 2003). Now that in the global economy customers determine the survival of a company, measuring patient’s satisfaction with health services plays an important role in increasing the responsibility and accountability commitment among health services providers (Woldeyohanes et al., 2015). Although significant investment has been devoted for medical and sanitary care, according to the World Health Organization, health improvements and care coverage and access have not been equally and fairly divided among and within countries so patients ‘demand for medical trip increases to receive satisfactory and effective services (WHO, 2000). Hospitals as an institution for providing health services should pave the way for understanding and respecting patients, their families, physicians, and other providers’ rights and responsibilities. Hospitals should be aware of care moral aspects and respect the role of a patient in treatment decision and other aspects of their care and be sensitive to individuals’ disability, culture, ethnicity, age and gender differences. Fakhredini et al. administered a study on Yazd medical tourists’ satisfaction and showed that service quality gap is meaningfully significant in three aspects; responsiveness, assurance and empathy. The study shows that having the appropriate equipment and proper treatment quality is in a better condition than other factors (Mirfakhradini, 2013). In a study conducted to evaluate patient satisfaction with nursing care, researchers showed that 82 percent of patients were satisfied with the services provided by nurses. The findings of the present study suggest an association between educational hospitals and the satisfaction level (Akhtari-Zavare et al., 2010). A study conducted by TC indicated that patient satisfaction average scores equaled 69.5 percent among 2953 hospital. Short stay of patients in hospitals has a positive relationship with the highest level of satisfaction. Hospitals in the top quarter of satisfaction had a higher level of performance and lower re-admission rates and less mortality than those hospitals in the lowest quarter. Hospitals benefiting high patient satisfaction were also generally rated with higher quality in all measures (Tsai et al., 2015). It is worth mentioning that hospitals are examples of organizations in which assessing customer satisfaction is considered as a very important issue because these organizations value customers’ needs, wishes and satisfaction greatly. Considering mentioned points, researchers conducted a study to eliminate existing research gaps and investigate patient satisfaction level in various medical, nursing, administrative, service and hoteling aspects.

## 2. Method

The present research is a descriptive survey in terms of methodology and time which was conducted in affiliated hospitals of Lorestan University of Medical Sciences in 2015 spring. Population was selected by simple random sampling. Since the population variance was obtained in earlier studies the variance of the study was considered 0.60. According to the following formula:


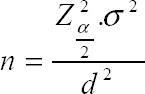


n = number of samples

S2 = Sample variance

D = estimation error

Z = standard unit normal variable value which equals 1.96 at confidence level of 95%.

For calculating the number of samples, confidence level, sample size, and error level were considered 95%, 0.6, and 0.023 relatively. The study sample size was 1,800. The data collection instrument is semi-structured questionnaire. The questionnaire has 62 general and specialized items. General items of the questionnaire contain questions about residence status, re-admissions, age, gender, marital status, education level and hospitalization method. Special items also included 55 questions related to medicine, nursing, administrative, service and hoteling. Each specific item has scaled on four points; satisfied, fairly satisfied, dissatisfied and O.K. The questionnaire validity is asserted by experts and professors remarks and its reliability is calculated using the Cronbach alpha for questionnaire. The validity is higher than the required amount of 0.70so it can be said that there internal components benefit an excellent correlation which shows acceptable validity and reliability of the data collection instruments. Two methods of descriptive statistics (including tables’ preparation, graphs and scattering indexes drawing) and inferential statistics (Pearson and Spearman correlation coefficients and multivariate regression analysis, Kolmogorov-Smirnov and partial correlation) are also used to analyze data.

## 3. Results

Findings 969 patients (54.4) were male and 812 (45.6) were female. In terms of marital status from 1781 patients 16.4% were single, 81.4 % were married and the rest of participants had other conditions. 38.2 % of patients were admitted for the first time, 40.5 % were admitted for the fourth or fifth times, and the remaining patients were admitted more than 5 times. Most of the patients (43.9) had a level of education lower than high school diploma and the lowest frequency was for PhD patients, 4 people. In the hospitalization ward most patients were in pediatric department of the hospital with the percentage of 21.5% and the lowest percentage was 2.2 patients from maternity ward. 69.5 were percent of patients under care in emergency ward, 14.9 percent of patients were in clinic while the rest of them were sent from clinics to be hospitalized. 74.5 percent of 1781 patients were native and the remainders were non native of the province (Table 1).

**Table 1:** Demographic characteristics of studied samples

	Variable	Percent	Frequency
**Admission times**	the first time	38.2	681
	4 to 5 times	40.5	722
	More than 5 times	21.2	378

**Age**	Man	54.4	969
	Woman	45.6	812

**Stay kind**	Native	74.5	1326
	Expatriates	25.5	455

**Marital status**	Single	16.4	292
	Married	81.4	1449
	Others	2.3	40

**Schooling level**	Less than high school diploma	43.9	781
	Diploma	28.1	501
	Associate Degree	10.8	192
	BS	14.4	257
	MA	2.6	46
	Ph.D.	0.3	4

**Hospitalization ward**	Domestic women	8.6	153
	Obstetrics	11.5	204
	Internal men	11	196
	Men Surgery	6.6	118
	Children	21.5	383
	Maternity	2.2	40
	Dialysis	10.1	179
	Emergency	8	149
	ICU	5.9	105
	CCU	14.7	261

**Kind of hospitalization**	Emergency	69.5	1237
	clinic	14.9	265
	Doctor’s office	15.7	279

According to Table 2, there isn’t any meaningful relationship between demographic characteristics of gender, marital status and satisfaction level in various fields. There exists a meaningful relationship between schooling factor and admission, medical, nursing, paramedical and hoteling and readmission wards which doesn’t exist between discharge ward and schooling status (Table 3). Among the studied hospitals the greatest mean and standard deviation of different factors in different hospitals are as follows: Hoteling (3.3±0.56), Poldokhtar Imam Khomeini hospital discharge (3.3±0.75), Elsheter Imam Khomeini hospital paramedical (2.3±0.67), Nurabad Ibn Sina Hospital paramedical (2.3± 0.58) and Poldokhtar Imam Khomeini Hospital paramedical (2.3± 0.60), in the area of nursing Elsheter Imam Khomeini Hospital (5.3±0.57), Nurabad Ibn Sina Hospital (5.3± 0.60) and Poldokhtar Imam Khomeini Hospital (5.3±0.48), in the medical field Poldokhtar Imam Khomeini Hospital (6.3±0.51) and in the admission field three hospitals; Elsheter Imam Khomeini hospital (5.3± 0.53), Nurabad Ibn Sina Hospital (5.3±0.53) and Poldokhtar Imam Khomeini hospital (5.3±0.51) (Table 2).

**Table 2:** The relationship between demographic characteristics and areas of satisfaction in studied hospitals

variable		Hoteling	discharge	paramedic	nursing	medical	admission
**Sex**	F amount	1.663	1.479	1.988	2.712	2.265	1.618
	Meaningful level	0.197	0.224	0.15	0.100	0.133	0.204

**marital status**	F amount	1.651	0.392	2.442	2.212	1.838	1.337
	Meaningful level	0.176	0.759	0.063	0.085	0.138	0.261

**Education**	F amount	2.329	1.010	4.709	7.633	2.740	1.082
	Meaningful level	0.030	0.417	0.000	0.000	0.012	0.000

**Readmission**	F amount	5.642	3.676	6.078	5.296	6.164	1.990
	Meaningful level	0.004	0.000	0.002	0.005	0.002	0.000

**Table 3:** Mean standard deviation and satisfaction percent of different aspects of satisfaction in studied hospitals

variable		admission	medical	nursing	paramedic	discharge	Hoteling	Total satisfaction
	Mean (SD)	0.63±3	1±2.2	0.82±2.8	0.63±3	0.83±2.8	0.79±2.2	
**Imam Khomeini Kuhdasht**	Percent satisfaction	60	44	56	60	56	44	53
**Imam Khomeini Elshter**	Mean (SD)	0.53±3.5	0.54±3.6	0.57±3.5	0.67±3.2	0.76±3.2	0.68±3.2	
	Percent satisfaction	70	72	70	64	64	64	67
**Imam Sadiq (AS) Aligudarz**	Mean (SD)	0.80±3	1±2.8	0.81±3	0.72±2.6	0.91±2.6	0.90±2.5	55
	Percent satisfaction	60	56	60	52	52	50	
**SHohadaye Ashayer**	Mean (SD)	0.72±3	0.80±3.1	0.76±3.2	0.74±2.8	0.87±2.9	0.80±2.7	59
**KHoram Abad**	Percent satisfaction	60	62	64	56	58	54	
**Imam Ali(AS)**	Mean (SD)	0.75±3.2	0.91±3.2	1±3.4	0.71±2.8	0.83±2.7	0.77±3	61
**Azna**	Percent satisfaction	64	64	68	56	54	60	
**Chamran martyr Broujerd**	Mean (SD)	0.77±2.9	1±2.7	0.085±3	0.65±2.7	0.84±2.7	0.76±2.5	55
	Percent satisfaction	58	54	60	54	54	50	
**Martyrs revolver Doroud**	Mean (SD)	0.86±3	0.98±3	0.88±3	0.75±2.9	0.88±3	0.82±2.7	59
	Percent satisfaction	60	60	60	58	60	54	
	Mean (SD)	0.71±3.2	0.83±3.1	0.73±3.3	0.61±2.7	0.87±2.8	0.75±2.7	59
**Imam Khomeini Broujerd**	Percent satisfaction	64	62	66	54	56	54	
**Martyrs madani**	Mean (SD)	0.63±3.2	0.84±3.2	0.79±3.4	0.67±2.9	0.82±3	0.65±2.9	62
**KHoram Abad**	Percent satisfaction	64	64	68	58	60	58	
**Ibn Sina**	Mean (SD)	0.53±3.5	0.68±3.5	0.60±3.5	0.58±3.2	0.84±3.2	0.80±3.2	67
**Nour Abad**	Percent satisfaction	70	70	70	64	64	64	
**Imam Khomeini**	Mean (SD)	0.51±3.5	0.51±3.6	0.48±3.5	0.60±3.2	0.75±3.3	0.56±3.3	68
**Pol** Dokhtar	Percent satisfaction	70	72	70	64	66	66	
**Martyrs Rahimi**	Mean (SD)	0.51±3.3	0.80±3	0.62±3.3	0.56±2.9	0.81±3	0.61±3	62
**KHoram Abad**	Percent satisfaction	66	60	66	58	60	60
**Total satisfaction**		64	62	65	85	59	57	61

According to the findings of the study, the highest percentage of satisfaction in all aspects (Hotelling, discharge, paramedics, nurses, medical and admission) is related to Elsheter Imam Khomeini hospital and Nurabad Ibn Sina Hospital with satisfaction level of 67%. The lowest level of satisfaction is also related to Kuhdasht Imam Khomeini hospital with satisfaction level of 53%. The overall satisfaction level for all hospitals equaled 61% (Table 3).

## 4. Discussion

Patients’ high satisfaction with hospital services reflects the durability of the hospital and patient’s satisfaction with hoteling, discharge, paramedics, nursing, medical and admission services is known as the most important predictor of patient’s overall satisfaction with the hospital (Buchanan et al., 2015)and it is attempted to determine patient outcomes associated with these services (Griffiths et al., 2012). This study is an attempt to assess the medical tourism satisfaction with received services in 2015 in Lorestan hospitals. According to the findings of the study, in the field of nursing and medicine the greatest satisfaction is achieved by Elsheter Imam Khomeini, Nurabad Ibn Sina and Poldokhtar Imam Khomeini hospitals equals 70%. The overall satisfaction rate of nursing and medicine in all studied hospitals equals 65% and 62% respectively. Matis et al. study conducted at University Hospital in Greece shows that patients’ satisfaction with medical services and nursing services equals89.7 and 86.4 percent respectively (Matis et al., 2009). Nemati et al. study also shows that 210 patients (57.6 percent) are satisfied with medical services and 252 patients (72.3 percent) are satisfied with nursing services. The highest percentage of clinical services satisfaction is because of answering patients’ questions and meeting their needs and the lowest level of patient satisfaction is with medical staff when needed (Nemati, 2015). Another study conducted in Babol by Nazari et al. represents a 75.94 percent satisfaction with medical services and 64.06 percent satisfaction with nursing services (Nazari, 2011). The results of the present study showed that patients’ satisfaction with doctors and nurses is more than other dimensions, and patients believe that a good medical staff is a determining factor in coming to a hospital. The impact of nursing care quality on patients’ satisfaction has been proven in several studies. High workload and not having enough nurses after the implementation of the health reform plan is one of the reasons why nursing services satisfaction is less than Matisse et al. study. Therefore in order to increase patient satisfaction, enough nurses should be available and they should try to attract more satisfaction. Mercuric et al. focus on the patients’ evaluation of nursing services, they show that the most important obstacle to effective care and satisfaction of nursing services can be introduced as nurse deficiency (Merkouris et al., 2004). There is 57 percent of satisfaction in hoteling area within Lorestan University of Medical Sciences affiliated hospitals which is less than satisfaction rate’s of other studies. The highest Hoteling satisfaction level specifies to Nurabad Ibn Sina Hospital and the lowest level of satisfaction is related to Kuhdasht Imam Khomeini hospital. Studies conducted by Kazemaini, A’zami and Ebrahimnia shows that hoteling satisfaction has equaled 85.3, 80, and 91.5 percent (Azami, 2003; Ebrahimniam, 2010; Kazemeini, 2011). Considering the results of other administered studies, it can be said that this study is in an intermediate place compared to other studies. According to the government program and the implementation of health reform plan, it is hoped that the continuation of this project helps the improvement of hotelling services quality in all hospitals of Ministry of Health and Medical Education, achievement of the minimum standard rank, determination of an identical definition for hoteling service quality, responsiveness to patients needs by hoteling services in hospitals. Therefore, issues such as increasing the number of hospital beds, improving the quality of the beds in different wards, patients food table, serum base, mattresses quality, blankets, sheets, pillows and patients’ clothes, the number and location of wheelchair and stretcher, beds’ Fittings, quality and variety of patients food, curtains and different types of separators, physical space and waiting room facilities for patients family members, human resources caring for the patient, hospitalization and emergency room heating and cooling systems, health services and bathroom and summons system can increase Hotelling satisfaction. Hospital admission office is generally the first point of patients contact with hospital. This section provides a communication channel between the service consumer and service provider, therefore satisfaction with these areas has a significant impact on the performance of hospitals. The hospital admission and discharge satisfaction respectively equals 64 percent and 59 percent in this study.

The results of the present study are somewhat close to Nemati’s results but very different from Ebrahimnia et al. results (Ebrahimniam, 2010; Nemati, 2015). Given the 8 scope of Ebrahimnia study, admission and discharge areas have been in an intermediate state. Improving admission and discharge process, enhancing wards information, reinforcement of units’ staffs’ human skills and decreasing waiting time should occur to increase patients’ satisfaction level. The results showed that there were no relationship between the gender characteristic and patient satisfaction with different areas of service provision, a result which confirms A’zami and Akbarzade but differs from Salehian and Masoud (Azami, 2003; Masoud & Taghizadeh, 2003). Also there aren’t any relationship between marital status and patients satisfaction with different areas of service provision which confirms Salehian and differs from Masoud’s study (Masoud, & Taghizadeh, 2003; Salehian, 2010). But there is a meaningful relationship between readmission and patients’ satisfaction with different areas of service provision; patients who are more satisfied with the provided services are more willing to readmit in the same hospital. This result is consistent with Masoud study but Salehian and Nemati stated that there is no relationship between readmission and satisfaction level. Previous experiences form expectations as a reason for readmission to hospitals. Therefore, people based on previously gained knowledge through hospital admissions moderated their expectations and thereby are more satisfied. The findings of the present study shows that more educated people are more satisfied with the services provided at the studied hospitals which is consistent with Salehian et al. study (Salehian, 2010). Given that the level of education changes a person’s rational and reasonable expectation of service delivery, it can be concluded that less educated people expect more and sometimes have irrational expectations because they do not have enough information about their own rights and staff responsibilities which in turn will lead to less satisfaction. This result has been inconsistent with Tadoqatyr and Bahrampour studies which indicates more educated people have different expectations: they are more sociable and aware of shortages and deficiencies and access more information resources compared to less educated people so their level of satisfaction is lower (Al-Doghaither, 2004; Bahrampour & Zolala, 2005). Generally, patients’ satisfaction with Puldokhtar Imam Khomeini Hospital is the highest satisfaction level among hospitals and equals 68 percent and Kuhdasht Imam Khomeini Hospital has the lowest satisfaction level, 53 percent. In general, the level of satisfaction in all areas and affiliated hospitals of Lorestan University of Medical Sciences is 61 percent which regarding the national and international studies is in an intermediate condition.

## 5. Conclusion

Despite the shortcomings that are observed in any of the areas, we should pay attention to patient-centered issues and patients needs and preferences to enhance health care quality level. Paying attention to patients’ preferences not only is morally appreciated but also will lead to the improvement of care provision and access to sustainable care practices. So it is needed to lead organization management toward customers preferences management because with this kind of management, customer is considered as a main factor and providing what he needs will be of great importance. Assessing patients’ satisfaction is one of the main indicators in measuring health system quality so this assessment and measurement need to be done on time and up to date and after that ways should be considered to remove identified shortcomings and deficiencies.
